# Favorable Genotypes of Type III Interferon Confer Risk of Dyslipidemia in the Population With Obesity

**DOI:** 10.3389/fendo.2022.871352

**Published:** 2022-06-16

**Authors:** Tiantian Xu, Bo Peng, Mengmeng Liu, Qingjing Liu, Junya Yang, Minli Qu, Na Liu, Lizhen Lin, Jing Wu

**Affiliations:** ^1^ Department of Endocrinology, Xiangya Hospital, Central South University, Changsha, China; ^2^ Hunan Engineering Research Center for Obesity and its Metabolic Complications, Xiangya Hospital, Central South University, Changsha, China; ^3^ National Clinical Research Center for Geriatric Disorders, Xiangya Hospital, Central South University, Changsha, China; ^4^ Keck School of Medicine, University of Southern California, Los Angeles, CA, United States; ^5^ School of Health and Related Research, University of Sheffield, Sheffield, United Kingdom; ^6^ National Engineering Research Center of Personalized Diagnostic and Therapeutic Technology, Xiangya Hospital, Central South University, Changsha, China

**Keywords:** type III interferon, *IFNL3*, dyslipidemia, obesity, single nucleotide polymorphism

## Abstract

**Background:**

Studies have indicated that the chronic state of inflammation caused by obesity leads to dyslipidemia. However, how the polymorphisms involved in these inflammatory pathways affect the lipid metabolism in people with obesity is poorly understood. We investigated the associations of inflammation-related gene polymorphisms with dyslipidemia in individuals with obesity living in China.

**Methods:**

This case–control study in a population with obesity involved 194 individuals with dyslipidemia and 103 individuals without dyslipidemia. Anthropometric indices of obesity, fasting plasma glucose, blood pressure, blood lipids, and C-reactive protein were evaluated. The genes we tested were *IL6* (interleukin 6), *IL6R* (interleukin 6 receptor), *FOXP3* (forkhead box P3), *TLR2* (toll-like receptor 2), *TLR4* (toll-like receptor 4), *IFNL3* (interferon lambda 3, formerly known as *IL28B*), and *IFNL4* (interferon lambda 4, formerly known as *IL29*). Polymorphisms were genotyped using matrix-assisted laser desorption/ionization-time of flight mass spectrometry.

**Results:**

There were significant differences in the allelic and genotype frequencies of *IFNL3* (*IL28B*) rs12971396, rs8099917, rs11882871, rs12979860, rs4803217 between non-dyslipidemia and dyslipidemia groups in people with obesity. These single nucleotide polymorphisms (SNPs) of *IFNL3* were highly linked (D′ and r > 0.90), so the result of one SNP could represent the result of other SNPs. For *IFNL3* rs12971396, people with the homozygous genotype (the major group) carried a higher risk of dyslipidemia than people with the heterozygous genotype (*P* < 0.001, OR = 4.46, 95%CI, 1.95–10.22).

**Conclusions:**

The favorable genotypes of type III interferon, which have a beneficial role in anti-virus function, were associated with dyslipidemia in a Chinese population with obesity. Type III interferon could have a pathologic role and confer risk of dyslipidemia in people with obesity and chronic inflammation.

## Introduction

Obesity has become an epidemic worldwide. In China, obesity has increased rapidly: the standardized prevalence of obesity and overweight in adults was 19.3% in 2013 and 25.6% in 2018 (1). Obesity and its metabolic complications take a major toll on public-healthcare systems and increase the health risks of individuals (2–4). One of the most common obesity-associated complications, dyslipidemia, plays a major part in the development of atherosclerosis and cardiovascular diseases, which cause high mortality and represent 32% of all global deaths and 38% of premature deaths under the age of 70 years (5, 6). Dyslipidemia has also been reported to be a risk factor for various types of cancer (7–9). Recent studies have revealed abnormalities of lipid metabolism in tumor cells to accelerate disease progression and to alter lipid metabolism, which can influence tumor growth (10, 11).

Obesity is accompanied by chronic low-grade inflammation. Studies have indicated that the chronic state of inflammation caused by obesity leads to dyslipidemia (12, 13). Obesity increases intestinal permeability, which results in higher circulating levels of lipopolysaccharide (LPS) (14). This intestinal-derived LPS can initiate an inflammatory cascade and induce cytokine secretion (15, 16). Such increased inflammation can impair cholesterol efflux, and inhibit triglycerides (TG) clearance by reducing lipoprotein lipase (LPL) activity and reducing very-low-density lipoprotein-associated apolipoprotein-E levels (17). Different lipid species due to obesity may also contribute to inflammation; free fatty acids can promote inflammation by binding indirectly to the toll-like receptors (TLRs) *TLR4* and *TLR2* (18).

Gene polymorphisms occur frequently in a population, and may explain individual variation in disease risk. Various studies have reported an association between dyslipidemia and single-nucleotide polymorphisms (SNPs) of genes which confer different susceptibility to diseases (19). However, most genetic studies have examined individual or combined components of metabolic indices (e.g., body mass index (BMI), blood pressure (BP), lipid metabolism, glucose metabolism) rather than to treat dyslipidemia as a binary trait. Dyslipidemia is affected by several factors (especially obesity), so it is important to control these confounders strictly to obtain convincing evidence of which gene polymorphisms are associated with dyslipidemia. Moreover, the genes identified in previous studies have mostly been involved in metabolism-related pathways (19–21). Whether polymorphisms of inflammation-related genes affect lipid metabolism is poorly understood. Taken together, due to the limitations of previous studies, the associations between polymorphisms of genes involved in proinflammatory pathways and dyslipidemia have not been identified. Better control of confounding factors (e.g., obesity and other metabolic indices) are needed to resolve this question.

To exclude the confounding effects of obesity, we focused specifically on a population with obesity and separated them into two groups by treating dyslipidemia as a binary trait. We took other confounders such as age, sex, and BP into consideration. We aimed to investigate the effect of gene polymorphisms involved in the inflammatory pathway [*IL6* (interleukin 6), *IL6R* (interleukin 6 receptor), *FOXP3* (forkhead box P3), *TLR2* (toll-like receptor 2), *TLR4* (toll-like receptor 4), *IFNL3* (interferon lambda 3, formerly known as *IL28B*), and *IFNL4* (interferon lambda 4, formerly known as *IL29*)] on lipid metabolism in Chinese individuals with obesity.

## Patients and Methods

### Study Cohort

We conducted a case–control study involving people of Chinese origin aged 18–50 years. Patients with obesity (BMI ≥28 kg/m^2^ and/or male waist circumference (WC) ≥90 cm or female WC ≥85 cm) were recruited from an Outpatient Clinic of Xiangya Hospital within Central South University (Changsha, China). “Dyslipidemia” was defined according to the 2016 Chinese guideline for the management of dyslipidemia published by the National Expert Committee (22). Dyslipidemia was diagnosed as triglycerides (TG) ≥1.56 mmol/L, and/or low-density lipoprotein-cholesterol (LDL-C) ≥3.19 mmol/L, and/or total cholesterol (TC) ≥5.6 mmol/L, and/or high-density lipoprotein-cholesterol (HDL-C) ≤0.88 mmol/L. Considering the effect of drug treatment on metabolic status, we only included patients who denied a history of drug treatment. We also excluded people with a history of diabetes mellitus or infectious diseases. Finally, 297 individuals with obesity (103 without dyslipidemia and 194 with dyslipidemia) were invited to participate in our study, all of whom agreed.

### Collection of Clinical and Biochemical Data and Genotyping

The clinical and biochemical data we collected were sex, age, height, weight, BMI, waist-to-hip ratio (WHR), systemic blood pressure (SBP), diastolic blood pressure (DBP), as well as levels of glycated hemoglobin (HbA1c), fasting plasma glucose (FPG), TC, TG HDL-C, LDL-C, aspartate aminotransferase (AST), alanine transaminase (ALT), free fatty acid (FFA), apolipoprotein A1 (APOA1), apolipoprotein B (APOB), and high-sensitive C-reactive protein (hs-CRP). Polymorphisms were genotyped using the MassARRAY^®^ matrix-assisted laser desorption ionization time-of-flight mass spectrometry system (Sequenom, San Diego, CA, USA) after polymerase chain reaction (PCR) amplification (23). Considering the pathophysiology of the dyslipidemia observed in inflammation, and based on our literature search, we selected genes and the SNPs involved in abnormal lipid metabolism caused by inflammation (12, 13, 18, 24–26). The polymorphisms we tested were: *IL6* rs10242595, rs1524107, rs2069845; *IL6R* rs2229238, rs4845617; *FOXP3* rs11091253, rs143012151, rs148307134, rs28935477, rs369083462, rs3761548, rs376158, rs782511378; *TLR2* rs1439166, rs3804099, rs3804100, rs1337, rs5743708; *TLR4* rs545307676, rs78293159, rs138158233, rs5030710; *IFNL3* (*IL28B*) rs12971396, rs4803219, rs8099917, rs11882871, rs12979860, rs4803217; *IFNL4* (*IL29*) rs373455854, rs748154928, rs747979593. The primers used for the SNPs are listed in **Table S1**. The PCR conditions were 94°C for 3 min; 40 cycles at 94°C for 30 s, 56°C for 25 s, 72°C for 30 s; final extension step at 72°C for 3 min. The detailed procedure has been documented (www.gene-quantification.de/sequenom/).

### Statistical Analyses

Statistical analyses were undertaken using SPSS 20.0 (IBM, Armonk, NY, USA). Tests for deviation from the Hardy–Weinberg equilibrium as well as allelic and genotypic frequencies were undertaken with SNPStats (www.snpstats.net/start.htm/). Analyses of linkage disequilibrium and haplotypes were also undertaken with SNPStats. Continuous variables are expressed as the mean ± standard deviation. Categorical variables are expressed as frequencies and percentages. Differences in clinical parameters and biological parameters were compared between groups by independent-sample *t*-tests (continuous variables) and chi-square tests (categorical variables). We adjusted for confounding factors (including sex and age) in the regression analysis. For a comparison of clinical characteristics, *P* < 0.05 (two-sided) was considered significant. For the analysis of SNPs, because we undertook statistical tests with multiple genes and multiple SNPs simultaneously, the *P*-value threshold was adjusted with the Bonferroni correction to be *P* < 0.003 (two-sided). The *post hoc* statistical-power test was done with the *post hoc* calculator (https://clincalc.com/stats/Power.aspx/).

## Results

### Clinical and Biochemical Characteristics of Participants

A total of 621 participants with obesity were recruited. After implementing the exclusion criteria, finally we evaluated 297 participants (103 individuals without dyslipidemia and 194 individuals with dyslipidemia). **Table 1** shows the clinical and biochemical characteristics of the study cohort (150 women and 147 men). There were no significant differences between the two groups with respect to sex, age, BMI, or WHR (*P* > 0.05). Levels of HbA1C and FPG were higher in the group of people with obesity who had dyslipidemia (*P* = 0.01 and 0.006, respectively). SBP and DBP were comparable between groups (*P* = 0.76 and 0.06, respectively). The typical lipid profile (TC, TG, LDL-C and HDL-C) of individuals in the non-dyslipidemia group was within the normal range whereas, in the dyslipidemia group, at least one of four parameters was abnormal. On average, levels of TC, TG, and LDL-C were significantly higher in the dyslipidemia group than in the non-dyslipidemia group (*P* < 0.001). In contrast, the HDL-C level was significantly lower in the dyslipidemia group compared with that in the non-dyslipidemia group (*P* < 0.001). The other lipid indices (FFA, APOA1, APOB) showed no significant difference between the non-dyslipidemia group and dyslipidemia group. Levels of ALT and AST were higher in people with obesity who had dyslipidemia (*P* < 0.01). The level of the inflammation marker hs-CRP was high in the non-dyslipidemia group (4.11 ± 3.41 mg/L) and dyslipidemia group (4.08 ± 4.15 mg/L). Hence, patients with obesity had chronic inflammation and were at a high risk of cardiovascular disease.

**Table 1:** Clinical and biochemical characteristics of the studied groups.

Characteristic	Obesity without dyslipidemia (n=103)	Obesity with dyslipidemia (n=194)	*P* value
Sex (F/M)	55/48	95/99	.47
Age (year)	32.22 ± 10.56	32.10 ± 8.64	.92
BMI (kg/m^2^)	33.70 ± 5.02	33.61 ± 5.06	.89
WHR	0.95 ± 0.06	0.95 ± 0.11	.69
HbA1C	5.75 ± 0.91	6.14 ± 1.54	.01
FPG (mmol/L)	5.73 ± 1.57	6.39 ± 2.59	.006
SBP (mmHg)	124.87 ± 19.03	125.66 ± 22.07	.76
DBP (mmHg)	85.55 ± 10.73	88.07 ± 11.18	.06
TC (mmol/L)	4.35 ± 0.58	5.43 ± 1.04	<.001
TG (mmol/L)	1.07 ± 0.29	2.83 ± 1.94	<.001
HDL-C (mmol/L)	1.38 ± 0.33	1.15 ± 0.26	<.001
LDL-C (mmol/L)	2.49 ± 0.46	3.59 ± 0.83	<.001
ALT (U/L)	37.96 ± 33.96	57.83 ± 44.59	.005
AST (U/L)	29.30 ± 16.55	40.33 ± 26.13	<.001
FFA (mmol/L)	0.61 ± 0.23	0.61 ± 0.23	.84
APOA1 (g/L)	1.29 ± 0.20	1.34 ± 0.24	.14
APOB (g/L)	1.54 ± 4.75	1.09 ± 0.26	.38
hs-CRP (mg/L)	4.11 ± 3.41	4.08 ± 4.15	.95

BMI, body mass index; WHR, waist-to-hip ratio; FPG, fasting plasma glucose; SBP, systemic blood pressure; DBP, diastolic blood pressure; TC, total cholesterol; TG, serum total triacylglycerol; HDL-C, high-density lipoprotein cholesterol; LDL-C, low-density lipoprotein cholesterol; ALT, alanine transaminase; AST, aspartate aminotransferase; FFA, free fatty acid; APOA1, apolipoprotein A1; APOB, apolipoprotein B; hs-CRP, high-sensitive C-reactive protein.

### Distributions and Associations of Alleles and Genotypes With Dyslipidemia in a Population With Obesity

We undertook the Hardy–Weinberg equilibrium test, analyses of allele and genotype frequencies, and linkage-disequilibrium analyses using SNPStats employing the function “Single SNP analysis”. The polymorphisms we screened were for *IL6*, *IL6R*, *FOXP3*, *TLR2*, *TLR4*, *IFNL3* (*IL28B*), and *IFNL4* (*IL29*). Polymorphisms of *IFNL3* (rs12971396, rs4803219, rs8099917, rs11882871, rs12979860, rs4803217) varied between the non-dyslipidemia group and dyslipidemia group (*P* < 0.05).

The allelic and genotypic frequencies of these single-nucleotide sites of *IFNL3* in the study cohort were consistent with the Hardy–Weinberg equilibrium (*P* > 0.05). **Table 2** shows the allele frequencies of six single-nucleotide sites of *IFNL3* (rs12971396, rs4803219, rs8099917, rs11882871, rs12979860, rs4803217) between people with obesity without dyslipidemia and cases with obesity with dyslipidemia. After using the Bonferroni correction, we adjusted the *P*-value threshold to *P* < 0.003. We included five SNPs of *IFNL3* (rs12971396, rs8099917, rs11882871, rs12979860, rs4803217) for analysis of multiple SNPs.

**Table 2:** Hardy–Weinberg equilibrium test and allelic frequencies.

SNP	Allele	Obesity without dyslipidemia (n=103)	Obesity with dyslipidemia (n=194)	*χ²*	*P* value
rs12971396	C	182 (88)	373 (96)	13.29	<.001
	G	24 (12)	15 (4)		
	Total (2n)	206	388		
	HWE *P*	0.35	>0.99		
rs4803219	C	183 (89)	370 (95)	8.92	.003
	T	23 (11)	18 (5)		
	Total (2n)	206	388		
	HWE *P*	0.61	>0.99		
rs8099917	T	181 (88)	373 (96)	14.65	<.001
	G	25 (12)	15 (4)		
	Total (2n)	206	388		
	HWE *P*	0.35	>0.99		
rs11882871	A	181 (88)	371 (96)	12.31	<.001
	G	25 (12)	17 (4)		
	Total (2n)	206	388		
	HWE *P*	0.35	>0.99		
rs12979860	C	181 (88)	371 (96)	12.31	<.001
	T	25 (12)	17 (4)		
	Total (2n)	206	388		
	HWE *P*	0.35	>0.99		
rs4803217	C	181 (88)	369 (95)	10.28	.001
	A	25 (12)	19 (5)		
	Total (2n)	206	388		
	HWE *P*	0.35	>0.99		

SNP, single-nucleotide polymorphism; P, Pearson’s P value; HWE, Hardy–Weinberg equilibrium. The results are presented as No. (%).

The allele and genotype distributions of these five single-nucleotide sites of *IFNL3* were nearly identical. Linkage-disequilibrium analysis (**Table S2**) indicated that these sites were highly correlated with each other (values of D′ and r were >0.90). The haplotype analysis also indicated that these SNPs of *IFNL3* were combined and inherited together (**Table S3**). Hence, we chose rs12971396 as a representative SNP for further analysis. With regard to the rs12971396 polymorphism of *IFNL3*, the C allele was the major allele in the non-dyslipidemia and dyslipidemia groups (88% and 96%, respectively). There was a significantly higher proportion of the C allele in the group with obesity with dyslipidemia than the group with obesity without dyslipidemia (χ² = 13.29, *P* < 0.001).

There were two genotypes of *IFNL3* rs12971396 in the study cohort: homozygotic and heterozygotic (**Table 3**). The homozygote contained two major alleles. The heterozygote contained one major allele and one minor allele. The proportion of the *IFNL3* rs12971396 CC genotype was significantly higher in the dyslipidemia group, whereas the CG genotype had a lower proportion (**Table 3**). These findings indicated that the CC genotype of rs12971396 was probably a risk factor for dyslipidemia in the population with obesity (OR = 3.57, 95%CI, 1.82–7.14). Taking into consideration confounding factors such as levels of HbA1C, FPG, AST, and ALT, which were not comparable between groups, we carried out binary logistic regression analysis of dyslipidemia and genotype (**Table 4**), setting those possible confounders as covariates. Results suggested that, after adjustment for the glucose metabolism and liver function, the rs12979860 CC genotype was a significant risk factor that led to dyslipidemia,

**Table 3:** Genotypic frequencies between obesity with/without dyslipidemia.

SNP	Genotype	Obesity without dyslipidemia (n=103)	Obesity with dyslipidemia (n=194)	OR (95%CI)	*P* value
rs12971396	C/C	79 (77)	179 (92)	3.57 (1.82~7.14)	<.001
	C/G	24 (23)	15 (8)		

SNP, single-nucleotide polymorphism; P, Pearson’s P value. The results are presented as No. (%).

**Table 4:** Binary logistic regression analysis of dyslipidemia and genotype.

SNP	*β*	SE*β*	OR	95% CI	*P* value
rs12971396					
HbA1C	0.050	0.207	1.05	0.70~1.58	.81
FPG	0.141	0.127	1.15	0.90~1.48	.27
ALT	0.016	0.009	1.02	0.99~1.03	.07
AST	0.005	0.014	1.01	0.98~1.03	.75
(CC vs. CG)	1.50	0.423	4.46	1.95~10.22	<.001

SNP, single-nucleotide polymorphism; FPG, fasting plasma glucose; ALT, alanine transaminase; AST, aspartate aminotransferase. HbA1C, FPG, ALT, AST and genotype are independent variables, group (non-dyslipidemia or dyslipidemia) is the binary dependent variable.

To suppress the interference of glucose metabolism upon gene polymorphisms, we excluded patients who had an abnormal level of HbA1C (>6.1%) or FPG (>5.6 mmol/L). Under this circumstance, the *IFNL3* rs12971396 allele and genotype frequencies between the two groups continued to show significantly different distributions. Further, the major genotypes carried an even higher risk for the development of dyslipidemia than the minor genotypes (OR = 5.56, *P* = 0.001) (**Table S4**).

**Figure 1:**
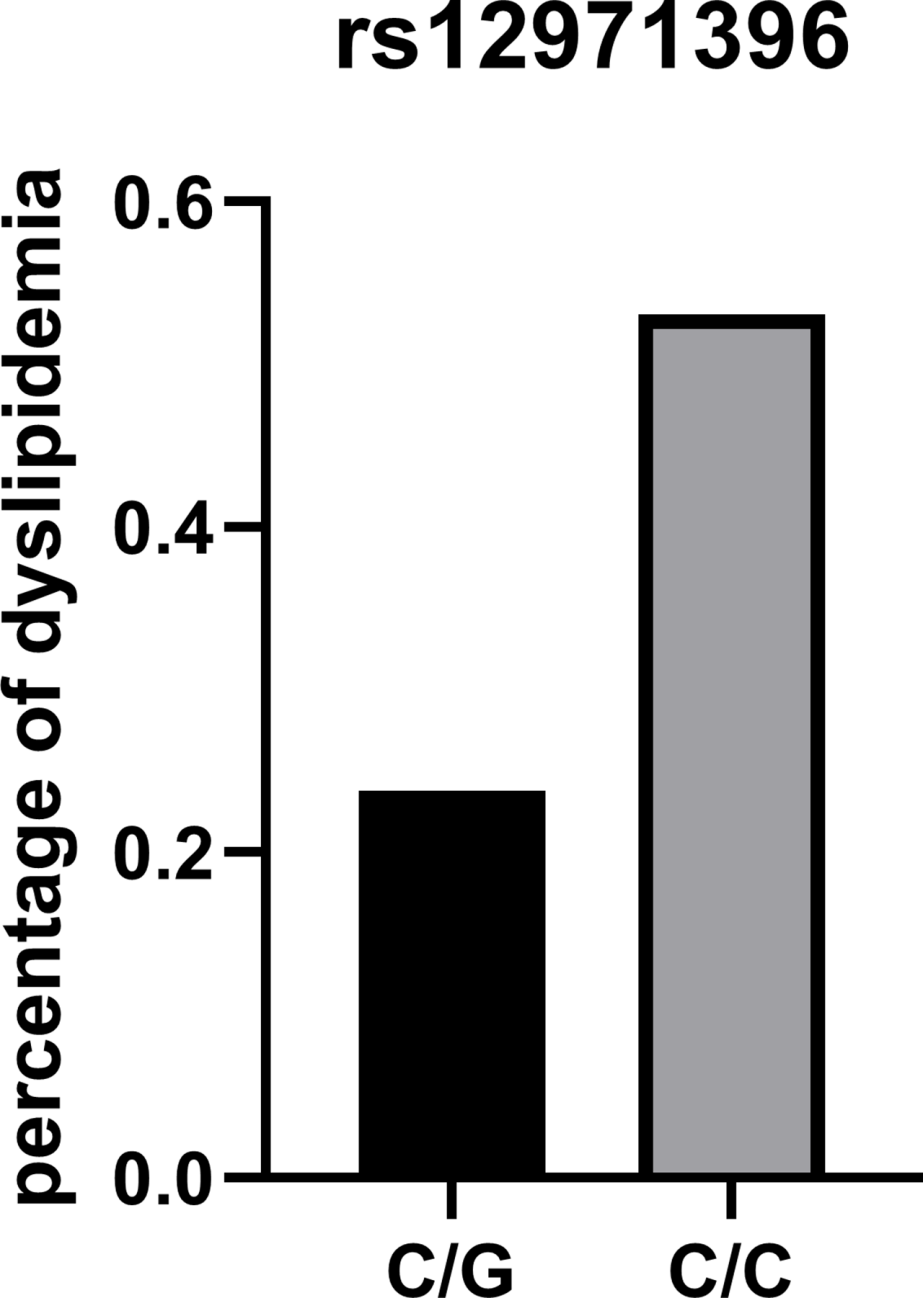
Percentage of dyslipidemia in different genotypes of IFNL3 rs12971396.

The percentage of people with dyslipidemia who had different *IFNL3* rs12971396 genotypes is presented in **Figure 1**. We normalized the sample number of the non-dyslipidemia group and dyslipidemia group to compare them. Overall, the percentage of people with dyslipidemia who had the homozygous genotype was 53%, which was much higher than the percentage of people with dyslipidemia who had the heterozygous genotype (24%). These data also suggested that the homozygotic genotype of these *IFNL3* loci was a risk factor for dyslipidemia in people who were obese.

The allele and genotype frequencies of other genes [*IL6*, *IL6R*, *FOXP3*, *TLR2*, *TLR4* and *IFNL4* (*IL29*)] did not show a significant difference between the two study groups (**Table S5**).

## Discussion

We discovered the polymorphisms of *IFNL3* (*IL28B*), an inflammation-related gene, to be associated with dyslipidemia in a population with obesity in China. The major allele and homozygotic genotypes were risk factors of dyslipidemia in individuals with obesity.

*IFNL3* (also known as *IFN-λ3*, formerly known as *IL28B*) is a type III IFN believed to have roles in modulation of the immune response during infection/inflammation (27). In contrast to type I IFNs (IFN-α and IFN-β), which are secreted by infected cells and can result in immunopathology during viral infections, type III IFN (IFNL) responses are restricted primarily to mucosal surfaces and are thought to confer antiviral protection without driving damaging proinflammatory responses (27, 28). Due to this ability to maintain antiviral activity and limit immunopathology, the development of IFNLs for clinical use as an alternative treatment to IFNα against viral infections (e.g., hepatitis C, coronavirus disease-2019) has been of interest recently (29–31). However, despite many studies indicating the benefits of type III IFNs in the protection and treatment for multiple infectious diseases, the long-term influence and pathophysiological role of IFNLs remain controversial. Several recent studies have shown that the role of IFNLs can be quite diverse and controversial depending on the timing, location, and level of the expression during chronic disease and severe disease (32–35).

The function of IFNLs and their ability to regulate immunity is further impacted by several SNPs. Many studies have identified *IFNL3* polymorphisms to be correlated with the outcome of hepatitis-C infection. Scholars have found that major alleles and favorable genotypes lead to higher expression of *IFNL3*, better response to therapy, and viral clearance in patients (36–42). Unlike other research limited to infectious diseases, we investigated people with obesity. We revealed that *IFNL3* favorable genotypes could increase the risk of dyslipidemia, which is opposite to its beneficial role observed in viral diseases.

In this study, we linked the polymorphisms of an inflammatory gene to dyslipidemia. Genetic studies on dyslipidemia have identified only the genes involved in metabolism-related pathways and not inflammation-related pathways, and their study groups had some important confounders, such as obesity and other metabolic abnormalities (19–21). To exclude those confounders, we recruited participants with obesity and divided them into two groups depending if they had dyslipidemia. The age, sex, BMI, and blood pressure were comparable between groups. We found significant associations of five highly linked SNPs of *IFNL3* (rs12971396, rs8099917, rs11882871, rs12979860, rs4803217) with dyslipidemia in the population with obesity. Upon comparison of allelic frequencies (**Table 2**) with *P* < 0.05 (two-sided) and frequency of the *IFNL3* rs12971396 C-allele of 88% in the non-dyslipidemia group and 96% in the dyslipidemia group, the power to detect an association between rs12971396 polymorphism and dyslipidemia reached 91.5%. Therefore, the significant differences in the gene polymorphisms in our study are convincing. To further adjust the effects of glucose metabolism and liver function, we undertook logistic regression analysis and confirmed that polymorphisms of *IFNL3* rs12971396 (as a representative locus) were associated with dyslipidemia in people with obesity. People with a homozygous genotype carried a higher risk (OR = 4.46, 95%CI, 1.95–10.22, *P* < 0.001) of dyslipidemia than those with a heterozygous genotype (**Table 4**). After excluding people with abnormal levels of FPG or HbA1C, the associations between *IFNL3* polymorphisms with dyslipidemia became even stronger (OR = 5.56, 95%CI, 1.75–16.67, *P* = 0.001) (**Table S4**). These results with strict control of confounding factors provided evidence that polymorphisms of *IFNL3* (an inflammation-related gene) were associated with dyslipidemia in individuals with obesity, which has not been reported previously. The major genotype of *IFNL3*, which can upregulate the IFLN3 expression (proved by previous research), could be a risk factor of dyslipidemia. These data suggest that *IFNL3* has a detrimental effect and could disturb lipid metabolism in people with obesity.

In summary, our findings shed new light on the nature of IFNLs and inspire rethinking of the pathophysiological role of IFNLs in clinical practice, especially in a population with obesity or with a low-grade inflammation state. In-depth study of how IFNLs affect lipid metabolism in people with obesity might uncover other meaningful features of the mechanism of action of IFNLs.

## Conclusions

We found *IFNL3* (*IL28B*) polymorphisms to be associated with dyslipidemia in a population with obesity in China. These results indicate that *IFNL3* could have a pathologic role in obesity and chronic inflammation.

## Data Availability Statement

The original contributions presented in the study are included in the article/**Supplementary Material**. Further inquiries can be directed to the corresponding author.

## Ethics Statement

The studies involving human participants were reviewed and approved by Xiangya Hospital of Central South University. The patients/participants provided their written informed consent to participate in this study.

## Author Contributions

Conceptualization: JW, TX, BP. Data curation: TX, BP, ML, QL, MQ, LL, NL. Formal analysis: TX, BP, ML, JY. Funding acquisition: JW, NL. Investigation: TX, BP, ML, JY. Supervision: JW, TX. Validation: JW, BP, ML, QL, JY, MQ, LL. Writing—original draft: TX, BP, JW. Writing—review & editing: all authors. All authors contributed to the article and approved the submitted version.

## Funding

The study was financially supported by the Key Research & Development Plan, Hunan, China (2020SK2066), the Project of Hunan Health Committee, Hunan, China (20201923), and the National Natural Science Foundation of China (82170849).

## Conflict of Interest

The authors declare that the research was conducted in the absence of any commercial or financial relationships that could be construed as a potential conflict of interest.

## Publisher’s Note

All claims expressed in this article are solely those of the authors and do not necessarily represent those of their affiliated organizations, or those of the publisher, the editors and the reviewers. Any product that may be evaluated in this article, or claim that may be made by its manufacturer, is not guaranteed or endorsed by the publisher.
