# Supplementary material for: Favorable Genotypes of Type III Interferon Confer Risk of Dyslipidemia in the Population With Obesity
Source: Front Endocrinol (Lausanne). 2022 Jun 16;13:871352. doi: 10.3389/fendo.2022.871352 (PMC9243353; doi:10.3389/fendo.2022.871352)
Supplement: Supplementary file 1 [file DataSheet_1.docx]

**Table S1. Primers sequence of IFNL3 SNP loci**

| **SNP loci** | **Primer sequence** |
| --- | --- |
| rs12971396_W1_9F | ACGTTGGATGTGAAGACCACGCTGGCTTTG |
| rs12971396_W1_9R | ACGTTGGATGGAAGAGCCTGGCTTAGCCC |
| rs12971396_W1_9U | ggaTTTGCGGCACCGAGG |
| rs4803219_W1_26F | ACGTTGGATGTGGGATGTAATTCCTGCCTG |
| rs4803219_W1_26R | ACGTTGGATGCTTTTCCTACATCAGCTGGG |
| rs4803219_W1_26U | ctgcGCTCCATGGGGCAGCTTTTATC |
| rs8099917_W1_8F | ACGTTGGATGCAATTTGTCACTGTTCCTCC |
| rs8099917_W1_8R | ACGTTGGATGACTGTATACAGCATGGTTCC |
| rs8099917_W1_8U | TTCCTTTCTGTGAGCAAT |
| rs11882871_W2_13F | ACGTTGGATGTCAGTCAATGGGTGCTGAG |
| rs11882871_W2_13R | ACGTTGGATGTCTCTTCCCTGTAGAAGGAC |
| rs11882871_W2_13U | ggGGATCCACTGTCTCAGATA |
| rs12979860_W2_11F | ACGTTGGATGTCGTGCCTGTCGTGTACTGA |
| rs12979860_W2_11R | ACGTTGGATGAGCGCGGAGTGCAATTCAAC |
| rs12979860_W2_11U | aagtGAGCTCCCCGAAGGCG |
| rs4803217_W2_20F | ACGTTGGATGCCAGTCATGCAACCTGAGAT |
| rs4803217_W2_20R | ACGTTGGATGATAAATAGCGACTGGGTGAC |
| rs4803217_W2_20U | GATTTTATTTATAAATTAGCCACTTG |

**Table S2. Linkage disequilibrium analysis**

**D' statistic**

|  | rs12971396 | rs8099917 | rs11882871 | rs12979860 | rs4803217 |
| --- | --- | --- | --- | --- | --- |
| rs12971396 | . | 0.9987 | 0.9987 | 0.9987 | 0.9987 |
| rs8099917 | . | . | 0.9987 | 0.9987 | 0.9987 |
| rs11882871 | . | . | . | 0.9993 | 0.9993 |
| rs12979860 | . | . | . | . | 0.9993 |
| rs4803217 | . | . | . | . | . |

**r statistic**

|  | rs12971396 | rs8099917 | rs11882871 | rs12979860 | rs4803217 |
| --- | --- | --- | --- | --- | --- |
| rs12971396 | . | 0.9853 | 0.9598 | 0.9598 | 0.936 |
| rs8099917 | . | . | 0.9729 | 0.9729 | 0.9488 |
| rs11882871 | . | . | . | 0.9993 | 0.9746 |
| rs12979860 | . | . | . | . | 0.9746 |
| rs4803217 | . | . | . | . | . |

**Table S3. IFNL3 haplotype association with dyslipidemia (n=297)**

| haplotype | rs12971396 | rs8099917 | rs11882871 | rs12979860 | rs4803217 | Freq | OR  (95% CI) | *P* value |
| --- | --- | --- | --- | --- | --- | --- | --- | --- |
| 1 | C | T | A | C | C | 0.926 | 3.57 (1.79~7.14) | <.001 |
| 2 | G | G | G | T | A | 0.066 | 1.00 |  |
| rare | * | * | * | * | * | 0.008 | * | * |

**Table S4. Genotypic frequencies between obesity with/without dyslipidemia – normal Hb1AC and FPG**

| **IFNL3**  **SNP** | **Genotype** | **Obesity without dyslipidemia (n=64)** | **Obesity with dyslipidemia**  (**n=71)** | **OR (95%CI)** | ***P* value** |
| --- | --- | --- | --- | --- | --- |
| rs12971396 | C/C | 48 (75) | 67 (94) | 5.56  (1.75~16.67) | .001 |
|  | C/G | 16 (25) | 4 (6) |  |  |

SNP, single-nucleotide polymorphism; *P*, Pearson’s *P* value. The results are presented as n (%).

**Table S5.** **Genotypic frequencies of other genes between obesity with/without dyslipidemia**

| **SNP** | **Genotype** | **Obesity without dyslipidemia (n=103)** | **Obesity with dyslipidemia**  (**n=194)** | **OR (95%CI)** | ***P* value** |
| --- | --- | --- | --- | --- | --- |
| *IL6* rs10242595 | A/A | 86 (83) | 165 (85) | 1.00 | 0.59 |
|  | A/G | 17 (17) | 28 (14) | 0.86 (0.45-1.66) |  |
|  | G/G | 0 (0) | 1 (1) | NA |  |
| *IL6* rs1524107 | T/T | 60 (58) | 118 (61) | 1.00 | 0.74 |
|  | C/T | 36 (35) | 60 (31) | 0.85 (0.51-1.42) |  |
|  | C/C | 7 (7) | 16 (8) | 1.16 (0.45-2.98) |  |
| *IL6* rs2069845 | A/A | 98 (95) | 190 (98) | 1.00  0.41 (0.11-1.57) | 0.19 |
|  | A/G | 5 (5) | 4 (2) |  |  |
| *IL6R* rs2229238 | C/C | 73 (71) | 148 (76) | 1.00  0.67 (0.38-1.18) | 0.22 |
|  | C/T | 28 (27) | 38 (20) |  |  |
|  | T/T | 2 (2) | 8 (4) | 1.97 (0.41-9.53) |  |
| *IL6R* rs4845617 | G/G | 20 (19) | 56 (29) | 1.00  0.57 (0.31-1.04) | 0.17 |
|  | A/G | 65 (63) | 104 (54) |  |  |
|  | A/A | 18 (18) | 34 (17) | 0.67 (0.31-1.45) |  |
| *FOXP3* rs148307134 | T/T | 95 (92) | 176 (91) | 1.00 | 0.62 |
|  | C/T | 6 (6) | 16 (8) | 1.44 (0.55-3.80) |  |
|  | C/C | 2 (2) | 2 (1) | 0.54 (0.07-3.89) |  |
| *FOXP3* rs3761548 | G/G | 80 (78) | 145 (75) | 1.00 | 0.74 |
|  | G/T | 16 (15) | 31 (16) | 1.07 (0.55-2.07) |  |
|  | T/T | 7 (7) | 18 (9) | 1.42 (0.57-3.54) |  |
| *TLR2* rs1439166 | T/T | 45 (44) | 92 (48) | 1.00 | 0.74 |
|  | C/T | 46 (45) | 77 (40) | 0.82 (0.49-1.36) |  |
|  | C/C | 12 (11) | 22 (12) | 0.90 (0.41-1.97) |  |
| *TLR2* rs3804099 | T/T | 48 (47) | 107 (55) | 1.00 | 0.21 |
|  | C/T | 49 (48) | 72 (37) | 0.66 (0.40-1.08) |  |
|  | C/C | 6 (5) | 15 (8) | 1.12 (0.41-3.07) |  |
| *TLR2* rs3804100 | T/T | 52 (50) | 117 (60) | 1.00 | 0.25 |
|  | C/T | 46 (45) | 68 (35) | 0.66 (0.40-1.08) |  |
|  | C/C | 5 (5) | 9 (5) | 0.80 (0.26-2.50) |  |
| *TLR2* rs1337 | C/C | 103 (100) | 193 (99) | 1.00 | 0.36 |
|  | C/G | 0 (0) | 1 (1) | NA |  |
| *IFNL4* rs748154928 | A/A | 102 (99) | 194 (100) | 1.00 | 0.14 |
|  | A/G | 1 (1) | 0 (0) | NA |  |

SNP, single-nucleotide polymorphism; *P*, Pearson’s *P* value. The results are presented as No. (%). Some genes SNPs (*FOXP3* rs11091253, rs143012151, rs28935477, rs369083462, rs376158, rs782511378; *TLR2* rs5743708; *TLR4* rs545307676, rs78293159, rs138158233, rs5030710; *IFNL4* (*IL29*) rs373455854, rs747979593) are not included in the table because only one genotype was detected in our study groups.
